# The impact of dietary linseed oil and pomegranate peel extract on broiler growth, carcass traits, serum lipid profile, and meat fatty acid, phenol, and flavonoid contents

**DOI:** 10.5713/ajas.18.0522

**Published:** 2019-01-04

**Authors:** Asmaa TY Kishawy, Shimaa A Amer, Mohamed E Abd El-Hack, Islam M Saadeldin, Ayman A Swelum

**Affiliations:** 1 Department of Nutrition and Clinical Nutrition, Faculty of Veterinary Medicine, Zagazig University, Zagazig 44511, Egypt; 2 Department of Poultry, Faculty of Agriculture, Zagazig University, Zagazig 44511, Egypt; 3 Department of Animal Production, College of Food and Agriculture Sciences, King Saud University, P.O. Box 2460, Riyadh 11451, Saudi Arabia; 4 Department of Physiology, Faculty of Veterinary Medicine, Zagazig University, Zagazig 44511, Egypt; 5 Department of Theriogenology, Faculty of Veterinary Medicine, Zagazig University, Zagazig 44511, Egypt

**Keywords:** Pomegranate Peel Extract, Linseed Oil, Meat, Phenols, Polyunsaturated Fatty Acid, Flavonoid

## Abstract

**Objective:**

The current study aimed to replace soybean oil in broiler diets with linseed oil, which is rich in omega-3 fatty acid supplemented with pomegranate peel extract (PPE) and measured its effect on broiler performance, carcass traits, lipid profile, as well as fatty acids composition, phenols and flavonoids content of broiler muscles and immunity of broiler chicks.

**Methods:**

A total of 300 1-day-old Cobb chicks were randomly allotted into six experimental groups, T1 fed on basal diet with soybean oil without any additives, T2 fed on basal diet with soybean oil with addition of 0.5 g/kg diet PPE, T3 fed on fed on basal diet with soybean oil with addition of 1 g/kg diet PPE, T4 fed on basal diet with linseed oil without any additives, T5 fed on basal diet with linseed oil with addition of 0.5 g/kg diet PPE and T6 fed on basal diet with linseed oil with addition of 1 g/kg diet PPE. The PPE supplementation with 0.05% improved final body weight with either soybean oil ration or linseed oil ration.

**Results:**

The PPE improved carcass dressing percentage in comparison with the control groups. Body fat levels decreased with increasing PPE levels, especially with a linseed oil diet. Replacing soybean oil with linseed oil decreased the total cholesterol and triacylglycerol levels in broiler serum. The PPE supplementation decreased serum total cholesterol levels and increased high-density lipoprotein cholesterol levels. The content of the breast muscle alpha linolenic acid improved after replacement of soybean oil with linseed oil in broiler diets. PPE supplementation increased the phenol and flavonoid content in broiler meat and increased lysozyme activity.

**Conclusion:**

Replacing soybean oil with linseed oil in broiler diets with the addition of PPE enriched muscle meat with omega-3 fatty acids and antioxidants and improved broiler immunity and their serum lipid profile.

## INTRODUCTION

Broiler chickens account for more than 90% of the total poultry population of the world [1]. The major parameters considered in the assessment of meat quality are appearance, juiciness, tenderness, flavour, and chemical composition of the meat [2]. Besides meat quality, the nutritional value of the meat and the health of the consumer should also be points of concern. Enrichment of broiler chicken muscles with polyunsaturated fatty acids (PUFAs), especially omega-3 and omega-6 fatty acids, is important for consumers, because PUFAs can reduce the risk of cardiovascular diseases, protect against atherosclerosis and coronary heart diseases by decreasing the cholesterol and low-density lipoprotein (LDL) levels in blood [3], and reduce platelet aggregation, which prevents excessive blood clotting [4]. Essential n-3 fatty acids reduce inflammation by decreasing the production of messenger chemicals such as cytokines [5] and increasing the activity of chemicals derived from endothelial cells that cause arteries to relax and dilate [6]. Flax seeds, which contain about 36% to 40% oil, are the richest (among crop plants) source of essential PUFAs in the human diet. Furthermore, they contain high levels of PUFAs, especially α-linolenic acid, unsaponified matter, total lipids, total tocopherols, total phenolics, and total flavonoids, sand therefore have high nutritional value [7]. Oxidative rancidity (OR) is one of the main causes of meat deterioration [8]. Besides producing an unpleasant odour, OR is responsible for the loss of flavour, texture, consistency, appearance, and nutritional value of the meat [9,10]. Antioxidants reduce lipid oxidation in meat and retain its PUFA content. However, the use of natural antioxidants to stabilize meat has gained much attention from consumers because they are considered to be safer than synthetic antioxidants [11]. Natural antioxidants, especially those derived from plants, have greater potential for improving the palatability, stability, and shelf-life of meat products and thereby increase consumer acceptability [11]. Pomegranate (*Punica granatum*) peel extract (PPE) is one example of a plant product containing a potential antioxidant.

Pomegranate peel is an attractive candidate for a nutritional supplement in animal feed. The PPE has high antioxidant capacity, considering its scavenging activity and protective activity against superoxide anions, hydroxyls, and peroxyl radicals. It also shows diverse pharmacological functions, including antioxidant activity [12,13]. Pomegranate contains 2.92±0.19 mg/100 g total phenols and 0.2% to 1.0% soluble phenols, showing remarkable antioxidant activity and significant health properties [14]. As a result of this high antioxidant activity, PPE has been shown to improve broiler performance and carcass dressing and decrease mortality [15]. The PPE also exerts antibacterial and antifungal effects [16], is hypocholesterolemic and hypolipidemic [17–19], and acts as an immuno-stimulant [20].

The current experiments were performed to compare the effect of diets containing soybean oil or linseed oil (PUFA-rich diets) supplemented with graded levels of PPE on broiler growth performance and carcass quality traits, especially body fat content, serum lipid profile, immunity, PUFA content, and the total phenol and flavonoid content of the breast muscles.

## MATERIALS AND METHODS

### Ethics statement

All animal studies were conducted in accordance with recommendations described in “The Guide for the Care and Use of Laboratory Animals” published by the Institutional Animal Care and Use Committee (IACUC) of Zagazig University.

### Animal management

Three-hundred 1-day-old chicks of a commercial meat type (COBB-500) obtained from a local hatchery were used in this study. On arrival, the chicks were weighed and randomly allocated to six equal treatment groups, each containing five replicates, with 10 chicks per replicate. A 2×3 factorial design consisting of two control groups was used: the first control group was fed a basal diet with soybean oil without any additives; the second control group was fed a basal diet with linseed oil without any additives. Both control diets were supplemented with a 0, 0.5, or 1 g PPE/kg diet. The birds were reared in a controlled environmental house with sawdust as litter at a density of 10 birds/m^2^. Continuous lighting was provided throughout the experiment. The starting temperature was 33°C, which decreased gradually by 2°C each week until it reached 21°C at week 6. Birds were vaccinated against Newcastle disease, infectious bronchitis, and gumboro diseases. Feed and water were provided *ad libitum* throughout the study. The experimental diet was formulated to meet the nutrient requirements set in a previous study [21] (Table 1). The experiment was divided into 3 stages; starter (from 0 to 10 days), grower (from 11 to 22 day), and finisher stage (from 23 to 42 day).

### Preparation of pomegranate peel extract

Pomegranate peels were collected after removal of the peel from local pomegranate fruit. The peels were dried in a hot-air oven at 40°C until a moisture content of approximately 8% (dry basis) was obtained. The moisture content was determined by oven drying at 105°C until a constant weight was achieved. The peels were ground and then used for extraction, as previously described [22].

### Antioxidant extraction

Samples of dried pomegranate peel powder were extracted by maceration in 70% ethanol (polar solvent with a dielectric constant of 24). The solvent was added to the dried and ground peel in a glass flask with a glass cover at a ratio of 10:1, beginning with a ratio of 4:1, and the residue was re-extracted 3 to 4 times until it was exhausted during maceration. Samples were placed in a thermostatic water bath shaker at room temperature for 48 h. The liquid extract was separated from solids by vacuum-enhanced filtration through Whatman No. 1 filter paper (Sigma-Aldrich Chemie GmbH, Steinheim, Germany). The alcohol was removed from the filtrate with a high-capacity evaporator (EYELA Rotary vacuum evaporator system). The ethanol-free extract was dried using a lyophilizer. The dried extracts were collected and weighed to calculate the percent extract yield [22,23]. The dried extracts were then kept in the dark at 4°C until use.

PPE%=total dried extract yield per kg/kg pomegranate peel×100

### Measurement of antioxidant activity and total phenol and flavonoid content of the pomegranate peel extract

The antioxidant activity of PPE was measured by determining the hydrogen-donating or radical-scavenging ability using a modified α,α-diphenyl-β-picryl hydrazyl (DPPH) radical method [24]. A 10-μL volume of 0.01 g/mL dried extract in methanol solution was added to 1 mL (500 μM) of DPPH solution and diluted to 25 mL with methanol. The solution was shaken vigorously with a vortex and incubated at room temperature (25°C±2°C) for 20 min. The decrease in absorbance at 517 nm was determined at the end of the incubation period with a spectrophotometer. The control sample was prepared as described above, but without any extract, and methanol was used as the blank. Radical-scavenging activity was expressed as the inhibition percentage (I%) and was calculated using the following formula [25]:

$$\text{I}\%=[({\text{A}}_{\text{C}}-{\text{A}}_{\text{S}})/{\text{A}}_{\text{C}}]\times 100$$

where A_C_ is the absorbance of the control reaction (containing all reagents except the test compound), and A_S_ is the absorbance of the test compound.

The total phenol and flavonoid contents were measured as previously described for PPE and sage leaf oil [12].

### Determination of the chemical properties of linseed oil and soybean oil

Refractive index was measured as described previously [26]; free fatty acid levels (FFA) were also measured in accordance with a previous study [27].

%FFA=AV×0.503

The acid value (AV) percentage was measured as previously described [27]; the iodine value (IV) was measured using a previously reported protocol [28], while the peroxide value was measured using a protocol described in another study [29].

### Analysis of fatty acids in experimental diets

The fatty acid composition of the experimental diets was analysed as follows:

#### Fat extraction from feed samples

Fat was extracted from the diet samples (3 samples per diet, 100 g) by maceration in hexane solvent (5:1) at room temperature for 24 h. Then, the samples were filtrated, and the solvent containing soluble fat was obtained and stored at 35°C until the hexane was removed. Fat samples were placed into Falcon tubes and stored in a refrigerator until analysis by gas chromatography for fatty acid composition.

#### Analysis of fatty acids

Saturated, unsaturated, and total fatty acid contents were determined in oil using the methyl esters boron trifluoride method [30]. The oil was saponified with sodium hydroxide in methanol. The fatty acids were methylated with boron trifluoride in methanol, extracted with heptanes, and analysed on a gas chromatograph with a flame ionization detector system, an autosampler, and an EZChrom integration system, with carrier gas (He) (ca. 25 Psi – air 450 mL – split 100 mL/min). The oven temperature was 200°C; the injector and detector temperature was 250°C.

### Evaluation of growth performance

Body weight (BW) and feed consumption were recorded at the end of each feeding stage. The total body weight gain (BWG) was calculated as (W2 – W1), where W1is the initial live weight (g) and W2 is the final live weight (g). The feed conversion ratio (FCR) was estimated as previously described [31], as there was no mortality as follows:

FCR=amount of feed consumed (g)/BWG (g)

Relative growth rate (RGR) was calculated using the following equation [32]:

$$\text{RGR}=\left[(\text{W}2-\text{W}1)/\frac{\left(\text{W}1+\text{W}2\right)}{2}\right]\times 100$$

Protein efficiency ratio (PER) was determined as described previously [33], as the grams of weight gain per unit of dietary protein consumed.

PER=live weight gain (g)/protein intake (g)

### Carcass traits

At the end of the experimental period (day-42), three birds from each group were selected, fasted overnight, weighed, and slaughtered using a sharp knife to complete bleeding. Feathers were then plucked and the birds were eviscerated and weighed to determine the dressing percentage. The dressed carcass, liver, gizzard, intestine, heart, spleen, bursa, and abdominal fat were weighed and the percent of live BW was calculated.

### Measurement of serum parameters

Total serum cholesterol level was measured as described previously [34]. Serum triacylglycerol concentration was determined calorimetrically in accordance with a previous study [35]. The serum high-density lipoprotein (HDL) level was determined colorimetric as described previously [34]. Serum LDL and very-low–density lipoprotein (VLDL) concentrations were calculated according to the method outlined in another study [36].

### Immunological studies of the blood

#### Lysozyme activity

Lysosomal activity in the serum was measured by agarose gel cell lysis assay, as previously described [37]. Briefly, lysoplates were prepared by dissolving 0.01% agarose in 0.0067 M phosphate-buffered saline (pH 6.3) at 100°C. When the agarose temperature reached 60°C to 70°C, 500 mg of a uniform suspension of *Micrococcus lysodeikticus* in 5 mL saline was added to 1 L of agarose and mixed well. The plates were then poured, using exactly 25 μL of each serum sample and standard lysozyme solutions in each well. Then, the plates were incubated at 28°C to 30°C for 12 to 18 h. and the ring diameters of the clear zones were measured. The diameters of sample-clear zones were plotted against the standard to determine the lysozyme concentration in units. The lysozyme content at day 1was subtracted from the lysozyme content at day 42 to yield the total increase in lysozyme content over the experimental period.

### Gas chromatographic analysis of breast muscle fat content

#### Extraction of lipids

Lipids were extracted using a solvent mixture of chloroform/methanol (2:1 v/v), which is suitable for quantitative extraction of lipids [38]. About 150 g of fresh breast muscle was used; samples were cut into small sections, macerated in solvent at a ratio of 3:1 solvent:muscle at room temperature for 48 h, and then filtrated; the filtrate was then kept in an oven at 35°C until all the solvent was evaporated. The extracted lipids were kept in Falcon tubes in a refrigerator until analysis.

#### Separation and identification of fatty acids

Fatty acids in oil extracted from muscles were identified as described for the ration [30].

### Determination of total phenol and total flavonoid contents in breast muscle

#### Extraction of the total phenol and flavonoid contents from muscles

The extraction of phenol and flavonoid compounds from muscles is influenced by the liquid-solid extraction conditions used to separate a soluble fraction from a permeable solid. A sample of about 25 g of fresh defatted muscle was cut into small pieces and then extracted by maceration in 70% ethanol. The solvent was added to the samples in a glass flask with a glass cover at a ratio of 6:1. During maceration, samples were placed in a thermostatic water bath shaker at room temperature for 48 h. The liquid extract was separated from solids by vacuum-enhanced filtration through Whatman No. 1 filter paper. The alcohol extracts of the muscles were kept in a refrigerator and subsequently analysed as previously described [22,23].

#### Total phenolic content

Total phenol content in the extracts was determined by the Folin–Ciocalteu method. Dried extract (0.05 g) was dissolved in 5 mL of methanol, or the filtrate was made up to 50 mL if used directly. Aliquots of samples (10 μL) were mixed with 2.5 mL of 10-fold-diluted Folin–Ciocalteu reagent and 2 mL of 7.5% sodium carbonate. The total volume of the mixture was adjusted to 25 mL using deionized water and allowed to stand for 30 min at room temperature before the absorbance was measured at 760 nm using a spectrophotometer. The total phenolic content in the extract was calculated and expressed as gallic acid equivalent (g/mL g dry mass) using a gallic acid (0.000 to 0.004 mg/mL) standard curve [12].

#### Total flavonoid content

The total flavonoid content was measured using a modified colorimetric method. Dried extract (0.05 g) was dissolved in 5 mL of methanol, or the filtrate was made up to 50 mL when used directly. A volume of 0.4 mL of the solution was transferred to a 25-mL flask containing 5 mL of 30% ethanol and mixed with 0.75 mL of 5% sodium nitrite for 5 min. Then, 0.75 mL of 10% aluminium nitrate was added. After 6 min, the reaction was stopped by adding 5 mL of 1 M sodium hydroxide. The mixture was further diluted with 30% ethanol up to 25 mL. The absorbance of the mixture was immediately measured at 510 nm. The flavonoid content was calculated and expressed as quercetin equivalents (g/100 g dry mass) using a quercetin (0.00 to 0.03 mg/mL) standard curve [12].

### Statistical analysis

All data were verified for normal distribution after transformation (ASIN). Statistical tests were performed using the general linear model multivariant procedure in SPSS version 21 for Windows (SPSS, Inc., Chicago, IL, USA). Post-hoc Tukey-B multiple-range tests were used to compare differences between the means at 5% probability. Standard error of the mean was calculated as the square root of the error mean square divided by the square root of the number of samples. The effects of replicates were analysed, but were not significant.

## RESULTS

Analysis of PPE for the percentage extract yield showed a value of 24.13%, total phenolic content was 600 mg/100 g powder, total flavonoid content was 8.5 mg/100 g powder, and radical-scavenging activity (I%) was 35.00 at 10 μg/mL.

The results of soybean and linseed oil analysis are shown in (Table 2) and, and the findings showed that linseed oil had higher FFA level, AV, and IV than soybean oil.

Fatty acids content of experimental diets are shown in (Table 3) and the finding showed that linseed oil containing diets show high content of alfa linolenic acid (omega-3) fatty acid.

### Growth performance

The effects of different dietary levels of PPE with different oil sources and their interactions on the growth performance of broiler chickens are shown in Table 4. Birds fed diets containing linseed oil without PPE and soybean oil without PPE had higher (p<0.05) BW and BWG than birds in the other groups, while group fed 0.1 PPE with soy oil had the lowest BW and BWG than birds in the other groups. The highest FCR (p<0.05) was recorded for the group fed soybean oil with 0.1% PPE during the starter period. During the grower period, birds fed soybean oil with 0.1% PPE showed lower BW (p<0.05) than birds in the other groups. The addition of 0.1% PPE to soybean oil diets resulted in decreased feed intake (FI) (p<0.05) compared with the other groups; decreased FI also observed in the linseed oil group without additives and with 0.1% PPE addition. The best FCR was recorded for group fed soybean oil without additives in grower period. Birds fed 0.1% PPE with soybean oil or linseed oil had the lowest BW (p<0.05) compared with the other groups; birds fed linseed oil with 0.1% PPE also had the lower BWG and FI, and the higher FCR, but not significant with control. The highest BW was recorded for groups fed 0.05% PPE with soybean oil and with linseed oil during the finisher period. In terms of overall performance, 0.05% PPE with soybean oil or linseed oil improved (p<0.05) the BW, BWG, FCR, RGR, and PER, compared with the values observed with 0.1% PPE with soybean oil or linseed oil (Table 5).

### Carcass traits

The effects of different dietary levels of PPE with different oil sources and their interactions on carcass traits of broiler chickens are shown in Table 6. Birds fed PPE at 0.05% and 0.1% with soybean oil or linseed oil showed significantly higher (p<0.05) carcass dressing percentages than those in the other groups. The group fed soybean oil without PPE had significantly higher (p<0.05) intestinal and liver percentage values than the other groups. Abdominal fat was significantly lower (p<0.05) in groups fed 0.1% PPE with soybean oil and linseed oil at 0.05% and 0.1%, compared with the other groups. The highest group in liver and intestinal % was that fed soybean oil without additives.

### Lipid profile

The effects of different dietary levels of PPE with different oil sources and their interactions on the lipid profile of broiler serum are shown in (Table 7). The addition of PPE to broiler ration, either with soybean oil or with linseed oil, at 0.05% or 0.1% resulted in decreased (p<0.05) total cholesterol, triacylglycerol, LDL cholesterol, and VLDL cholesterol levels, while the HDL cholesterol concentration increased (p<0.05) in broiler serum. Generally replacing soybean oil with linseed oil resulted in decreased (p<0.05) total cholesterol, triacylglycerol, LDL cholesterol, and VLDL cholesterol levels, while the HDL cholesterol levels increased (p<0.05).

### Fatty acid content in boiler breast muscles

The effects of different dietary levels of PPE with different oil sources and their interactions on the fatty acid content of broiler breast muscles is shown in Table 8. Addition of 0.1% PPE to linseed oil ration had recorded the highest level of alpha linolenic fatty acid comparing to other experimental group. Generally, replacing soybean oil with linseed oil increased precipitation (p<0.05) level of alpha linolenic fatty acid (omega-3) in broiler breast muscle and decreased (p<0.05) the oleic acid levels in broiler breast muscle.

### Antioxidant status of broiler breast muscles and bird immunity

The effects of different dietary levels of PPE with different oil sources and their interactions on the antioxidant status of breast muscles and the immunity of broiler chickens are shown in Table 9. The addition of PPE to broiler ration, either with soybean oil or linseed oil, at 0.05% or 0.1% increased (p<0.05) the total phenol and flavonoid content in muscle; their levels in muscle increased with increasing levels of PPE in the diet.

Supplementation of soybean oil or linseed oil with 0.1% PPE resulted in improved serum lysozyme activity and increased the percentage of the bursa of Fabricius and thymus gland to live BW (Table 6), indicating improved bird immunity.

## DISCUSSION

Poultry meat and its products have a vast consumer market and contribute significantly to the supply of good-quality animal protein, vitamins, and minerals [39]. Enrichment of broiler chicken muscles with PUFAs, especially omega-3 and omega-6 fatty acids, improves meat quality for the consumer and enhances human health [3]. Analysis of PPE for total phenolic content was 600 mg/100 g powder; total flavonoid content was 8.5 mg/100 g powder, and radical-scavenging activity (I%) was 35.00 at 10 μg/mL. The high content of antioxidant in pomegranate peel was enforced by the result of [40] who found that pomegranate peel had high antioxidant activity than leaves also, after characterization of the extract from peel he found about thirty eight poly phenolics compound, which means high antioxidant content.

The results of the current study showed that the addition of 0.05% PPE to poultry diets improved the final BW, total gain, FCR, RGR, and PER in both soybean oil diets and linseed oil diets, while with 0.1% PPE supplementation, all of those parameters decreased in comparison with the values in the control diets. The interactive effect between linseed oil and PPE in decreasing final BW may be due to linseed oil had decreased fat precipitation in birds body as reported by [41]. Also the findings from a previous study [42] have shown that rats fed high-fat diets containing pomegranate leaf extract exhibited decreased obesity through inhibition of pancreatic lipase, which suppressed lipid digestion and absorption, thus decreasing BW and weight gain. Similarly, increased levels of pomegranate peel in rabbit rations have reported to decrease animal performance and weight gain (Fayed, Azoz, Zedan, and Basyony, 2012). The results of the present study indicate that birds fed PPE at 0.05% and 0.1% with soybean oil or linseed oil had higher carcass dressing percentages than those in the other groups; the group fed soybean oil without PPE had higher intestinal and liver percentages than the other groups. Abdominal fat level was lower in the groups fed 0.1% PPE with soybean oil and linseed oil and in those fed linseed oil with 0.05% PPE compared with the other groups. The current results are consistent with those reported previously [23,42], which showed that pomegranate peel or extract or pomegranate leaf extract had an inhibitory effect on lipid metabolism due to the hypocholesterolemic and hypolipidemic effect in rats fed high-fat diets, thus decreasing body fat precipitation. In the current study, replacing soybean oil with linseed oil resulted in decreased total cholesterol, triacylglycerol, LDL cholesterol, and VLDL cholesterol levels and increased HDL cholesterol levels in broiler serum. Moreover, the addition of PPE to broiler ration, either with soybean oil or with linseed oil, at 0.05% or 0.1% resulted in decreased total cholesterol, triacylglycerol, LDL cholesterol, and VLDL cholesterol levels and increased HDL cholesterol levels in broiler serum. A previous study [43] investigated the effect of replacing soybean oil with linseed oil on the lipid profile of broiler chicks and found that linseed oil results in a decrease in total cholesterol and triacylglycerol levels. Consistent with the findings of previous studies [15,19,23,42], pomegranate was found to exert hypocholesterolemic and hypolipidemic effects and interfere with lipid digestion, absorption, and metabolism. The effects of different dietary levels of PPE with different oil sources on the fatty acid content of broiler breast muscles was clarified as follows: replacing soybean oil with linseed oil resulted in an increased precipitation level of omega-3 fatty acids such as alpha-linolenic acid in broiler breast muscle, and decreased the levels of oleic acid in broiler breast muscle. Within the range data reported previously [44], inclusion of linseed oil in broiler diets from 25 to 40 days of age increased the muscle content of alpha-linolenic acid. The addition of PPE to linseed oil-containing diets retained the PUFAs of the oil and improved the precipitation in broiler muscles; this effect may be attributed to the high antioxidant activity of methanolic extracts of pomegranate peel and their preservative effect on PUFAs in linseed oil, which prevents their oxidation [24]. The addition of PPE to broiler rations, either with soybean oil or linseed oil, at 0.05% or 0.1% resulted in increased total phenol and flavonoid content in muscles, and their levels in muscles increased with increasing levels of dietary PPE. The present study showed that PPE supplementation resulted in a total phenolic content of 600 mg/100 g powder, total flavonoid content of 8.5 mg/100 g powder, and radical-scavenging activity (I%) of 35.00 at 10 μg/mL. The high phenol and flavonoid content may be the cause of increased precipitation in broiler muscles. Supplementation with 0.1% PPE and soybean oil or linseed oil resulted in improved lysozyme activity in broiler serum and increased the percentage of bursa of Fabricius and thymus gland relative to the live BW. Our results are consistent with those obtained in a previous study [45] that assessed the effect of *Punica granatum* L. (Punicaceae) fruit rind powder (PGFRP) 100 mg/kg provided orally as an aqueous suspension on cell-mediated and humoral components of the immune system in rabbits. PGFRP elicited an increase in the antibody titre to typhoid-H antigen. It also enhanced the inhibition of leukocyte migration in the leukocyte migration inhibition test, and the duration in a delayed hypersensitivity test with purified protein derivative, confirming its stimulatory effect on cell-mediated immune response.

## CONCLUSION

Addition of 0.05% PPE to soybean oil or linseed oil with the in broiler rations resulted in improved broiler BW, total BWG, and FCR. Supplementation of diets with 0.1% PPE decreased serum lipid and abdominal fat levels and increased flavonoids in broiler muscles. Replacing soybean oil with linseed oil in broiler diets with the addition of PPE resulted in broiler muscles enriched with PUFAs, especially omega-3 fatty acid, and antioxidants.

## Figures and Tables

**Table 1:** Contents and chemical composition of the diets

Items	Starter		Grower		Finisher	
		
	Soy oil diet	Linseed oil	Soy oil diet	Linseed oil	Soy oil diet	Linseed oil
Yellow corn	58	58	62	62	63.5	63.5
Soybean meal, 48%	34.66	34.66	28.2	28.2	25	25
Corn gluten, 60%	1.5	1.5	3	3	3	3
Wheat bran	-	-	1.1	1.1	1.8	1.8
Soy oil	2	-	2	-	3.26	-
Linseed oil	-	2	-	2	-	3.26
Calcium carbonate	1	1	1	1	1	1
Dicalcium phosphate	1.8	1.8	1.7	1.7	1.5	1.5
Common salt	0.3	0.3	0.3	0.3	0.3	0.3
Premix1)	0.3	0.3	0.3	0.3	0.3	0.3
DL-methionine, 98%	0.18	0.18	0.14	0.14	0.11	0.11
Lysine, HCl, 78%	0.16	0.16	0.16	0.16	0.13	0.13
Toxenil	0.1	0.1	0.1	0.1	0.1	0.1
Calculated composition2) (%)						
ME (kcal/kg)	3,047.52	3,047.52	3,090.10	3,090.10	3,178.57	3,178.57
CP	22.17	22.17	20.43	20.43	19.08	19.08
EE	4.50	4.50	4.62	4.62	5.88	5.88
CF	2.64	2.64	2.64	2.64	2.64	2.64
Ca	0.96	0.96	0.93	0.93	0.87	0.87
Available P	0.47	0.47	0.44	0.44	0.40	0.40
Lysine	1.36	1.36	1.19	1.19	1.09	1.09
Methionine	0.54	0.54	0.49	0.49	0.45	0.45

ME, metabolic energy; CP, crude protein; EE, ether extract; CF, crude fibre; and Ca, calcium.

1) Muvco premix: Each 1 kg contains vit. A 4,000,000 IU, vit. D_3_ 800,000 IU, vit. E 4 g, vit. K_3_ 400 mg, vit. B_1_ 400 mg, vit. B_2_ 2 g, vit. B_6_ 0.6 g, pantothenic acid 4 g, vit. B_12_ 4 mg, niacin 12 g, folic acid 400 mg, biotin 20 mg, Fe 12 g, Mn 24 g, Cu 1.6 g, I 120 mg, Co 40 mg, Se 40 mg, and Zn 20 g.

2) According to (Vantress [21]).

**Table 2:** Chemical analysis of soybean oil and linseed oil

Variables	Soybean oil	Linseed oil
Refractive index (RI 60)	1.459	1.464
Refractive index (RI 20)	1.473	1.478
Free fatty acids (FFA %)	0.19	1.35
Acid value (AV %)	0.37	2.68
Iodine value (IV %)	117.42	158.04
Peroxide value (PV meq/kg)	2.70	2.75

**Table 3:** Fatty acid composition (% of total fatty acids) of the diets used in the experiments

Fatty acids	Experimental diets					

	Soybean oil diets			Linseed oil diets		
	
	Starter	Grower	Finisher	Starter	Grower	Finisher
Palmitic acid C16:0	10.83±0.173	10.57±0.167	10.37±0.152	8.31±0.133	8.32±0.133	8.42±0.142
Stearic acid C18:0	3.53±0.398	3.41±0.398	3.51±0.258	3.69±0.393	3.50±0.375	3.65±0.336
Oleic acid C18:1n9	20.64±0.826	20.97±0.837	20.69±0.845	18.83±0.698	19.58±0.687	18.78±0.642
Palmitoleic acid C16:1n7	4.52±0.013	4.64±0.021	5.02±0.011	4.74±0.013	4.49±0.014	4.72±0.014
Linoleic acid C18:2n6	51.91±1.276	50.57±1.241	51.44±0.947	23.56±0.676	24.24±0.693	24.34±0.543
Alpha-linolenic acid C18:3n3	3.22±0.017	3.15±0.017	3.48±0.037	33.84±0.185	32.92±0.167	32.52±0.147
Gamma-linolenic acid C18:2n6	0.94±0.017	1.14±0.017	1.12±0.023	0.67±0.017	0.73±0.017	0.75±0.027
Other fatty acids	4.41±0.51	5.55±0.20	4.39±0.27	6.36±0.72	6.22±0.65	6.82±0.55

**Table 4:** Effects of different dietary levels of pomegranate peel extract with different oil sources and their interactions on body weight, body weight gain, feed intake, and feed conversion ratio during the experimental periods

Items		Int. wt. (g)	Starter (0 to 10 d)				Grower (11 to 22 d)				Finisher (23 to 42 d)			
		
			BW (g)	BWG (g)	FI (g)	FCR	BW (g)	BWG (g)	FI (g)	FCR	BW (g)	BWG (g)	FI (g)	FCR
Oils														
Soybean oil		46.60	360.38^b^	313.78	413.70^b^	1.33	1,252^b^	891.87	1,211	1.36	2,162	910	1,612	1.78
Linseed oil		46.13	382.02^a^	335.88	424.11^a^	1.26	1,290^a^	908.07	1,213	1.34	2,215	925	1,556	1.71
p-value		0.45	0.00	0.15	0.00	0.15	0.03	0.142	0.854	0.122	0.136	0.655	0.107	0.181
PPE level %														
0		46.80	393.34^a^	347.54^a^	435.62^a^	1.25^b^	1,275^a^	882.53^b^	1,228^b^	1.40^a^	2,169^b^	893.88^b^	1,567^b^	1.77^ab^
0.05		46.60	368.38^b^	322.78^b^	409.29^b^	1.27^b^	1,300^a^	931.63^a^	1,245^a^	1.34^b^	2,328^a^	1,028.5^a^	1,678^a^	1.64^b^
0.1		47.70	351.88^c^	304.18^c^	411.80^b^	1.36^a^	1,237^b^	885.75^b^	1,164^a^	1.31^b^	2,068^c^	830.88^b^	1,507^b^	1.83^a^
p-value		0.15	0.00	0.01	0.00	0.003	0.001	0.001	0.00	0.001	0.00	0.00	0.001	0.025
Interaction effects														
Soybean oil	0	46.80	389.89^ab^	344.09^a^	434.60	1.26^b^	1,247^ab^	857.86^c^	1,275^a^	1.49^a^	2,104^bc^	856.75^bc^	1,567^ab^	1.84^ab^
	0.05	46.60	359.25^c^	312.65^c^	400.92	1.29^b^	1,301^a^	942.50^a^	1,236^ab^	1.31^b^	2,284^a^	982.25^ab^	1,686^a^	1.72^ab^
	0.1	47.40	332.00^d^	284.60^d^	405.53	1.42^a^	1,207^b^	875.25^bc^	1,123^d^	1.28^b^	2,099^bc^	891.75^bc^	1,581^ab^	1.79^ab^
Linseed oil	0	46.80	396.80^a^	351.00^a^	436.60	1.24^b^	1,304^a^	907.20abc	1,180^c^	1.30^b^	2,235^ab^	931.00abc	1,567^ab^	1.70^ab^
	0.05	46.60	377.50^bc^	332.90^ab^	417.67	1.26^b^	1,298^a^	920.75^ab^	1,255^a^	1.36^b^	2,373^a^	1,074.75^a^	1,669^a^	1.56^b^
	0.1	48.00	371.50^bc^	323.75^bc^	418.06	1.29^b^	1,268^a^	896.25abc	1,205^bc^	1.35^b^	2,038^c^	770.00^c^	1,433^b^	1.87^a^
p-value		0.21	0.01	0.03	0.11	0.04	0.04	0.04	0.00	0.00	0.008	0.03	0.04	0.04
Pooled SEM		0.31	1.94	3.50	1.90	0.01	5.83	5.33	4.87	0.01	17.11	16.58	16.48	0.03

BW, body weight; BWG, body weight gain; FI, feed intake, FCR, feed conversion ratio; PPE, pomegranate peel extract; SEM, standard error of the mean.

a–d Means within the same column carrying different superscripts are significantly different at p≤0.05.

**Table 5:** Effects of different dietary levels of pomegranate peel extract with different oil sources and their interactions on broiler overall growth performance

Items		BW (g)	BWG (g)	FI (g)	FCR	RGR	PER
Oils							
Soybean oil		2,162	2,115	3,237	1.53^b^	191.53	3.34^b^
Linseed oil		2,215	2,169	3,194	1.48^a^	191.79	3.46^a^
p-value		0.136	0.134	0.331	0.003	0.165	0.002
PPE level %							
0		2,169^b^	2,123^b^	3,231^a^	1.52^a^	191.71^b^	3.36^b^
0.05		2,328^a^	2,282^a^	3,333^a^	1.46^b^	192.30^a^	3.50^a^
0.1		2,068^c^	2,020^c^	3,083^b^	1.53^a^	190.96^c^	3.35^b^
p-value		0.00	0.00	0.00	0.005	0.00	0.003
Interaction effects							
Soybean oil	0	2,104^bc^	2,058^bc^	3,277	1.59^a^	191.47^a^	3.21^d^
	0.05	2,284^a^	2,237^a^	3,323	1.49^bc^	191.98^a^	3.44^bc^
	0.1	2,099^bc^	2,051^bc^	3,111	1.52abc	191.14^a^	3.38bcd
Linseed oil	0	2,235^ab^	2,189^ab^	3,185	1.46^bc^	191.95^a^	3.51^ab^
	0.05	2,373^a^	2,328^a^	3,342	1.44^c^	192.62^a^	3.55^a^
	0.1	2,038^c^	1,990^c^	3,056	1.54^ab^	190.79^b^	3.33^cd^
p-value		0.04	0.04	0.572	0.004	0.047	0.002
Pooled SEM		17.11	17.18	21.66	0.01	0.09	0.02

BW, body weight; BWG, body weight gain; FI, feed intake, FCR, feed conversion ratio; RGR, relative growth rate; PER, protein efficiency ratio; PPE, pomegranate peel extract; SEM, standard error of the mean.

a–d Means within the same column carrying different superscripts are significantly different at p≤0.05.

**Table 6:** Effects of different dietary levels of pomegranate peel extract with different oil sources and their interactions on broiler chickens’ carcass traits

Items		Dressed %	Intestine %	Liver %	Gizzard %	Heart %	Abdominal fat %	Spleen %	Bursa %	Thymus %
Oils										
Soybean oil		68.30	5.09^a^	2.11^a^	1.90	0.36	1.08^a^	2.45	2.17^b^	1.56^b^
Linseed oil		68.38	4.28^b^	1.76^b^	1.94	0.38	0.84^b^	2.56	3.35^a^	2.11^a^
p-value		0.890	0.001	0.006	0.583	0.112	0.00	0.298	0.00	0.006
PPE level %										
0		66.34^b^	5.35^a^	2.34^a^	2.05^a^	0.40^b^	1.08^a^	2.55	2.29^c^	1.61^c^
0.05		69.10^a^	4.47^b^	1.74^a^	1.90^a^	0.40^a^	0.97^b^	2.44	2.77^b^	1.86^b^
0.1		69.60^a^	4.24^b^	1.72^b^	1.82^b^	0.31^c^	0.84^c^	2.52	3.22^a^	2.05^a^
p-value		0.030	0.03	0.00	0.047	0.00	0.00	0.651	0.00	0.03
Interaction effects										
Soybean oil	0	65.68^c^	5.93^a^	2.72^a^	1.87^ab^	0.39	1.14^a^	2.49	1.88^d^	1.37^e^
	0.05	68.65^a^	4.84^b^	1.80^b^	1.92^ab^	0.38	1.19^a^	2.37	2.21^cd^	1.59^d^
	0.1	69.27^a^	4.50^b^	1.82^b^	1.92^ab^	0.31	0.91^bc^	2.49	2.43^cd^	1.73^cd^
Linseed oil	0	66.99^b^	4.76^b^	1.97^b^	2.23^a^	0.42	1.04^ab^	2.61	2.70^c^	1.85^c^
	0.05	69.54^a^	4.09^b^	1.68^b^	1.88^ab^	0.41	0.75^c^	2.51	3.32^b^	2.12^b^
	0.1	69.92^a^	3.98^b^	1.63^b^	1.71^b^	0.31	0.76^c^	2.55	4.02^a^	2.37^a^
p-value		0.04	0.001	0.00	0.013	0.350	0.004	0.946	0.034	0.03
Pooled SEM		0.28	0.11	0.06	0.04	0.01	0.02	0.05	0.06	0.02

PPE, pomegranate peel extract; SEM, standard error of the mean.

a–d Means within the same column carrying different superscripts are significantly different at p≤0.05.

**Table 7:** Effects of different dietary levels of pomegranate peel extract with different oil sources and their interactions on broiler chickens’ serum lipid profile (mg/dL)

Items		Cholesterol	Triacylglycerol	HDL-cholesterol	VLDL-cholesterol	LDL-cholesterol
Oils						
Soybean oil		186.21^a^	83.66^a^	65.25^b^	16.73^a^	137.69^a^
Linseed oil		163.97^b^	58.18^b^	70.86^a^	11.64^b^	104.75^b^
p-value		0.00	0.00	0.01	0.00	0.00
PPE level %						
0		207.45^a^	80.02^a^	57.74^c^	16.00^a^	165.71^a^
0.05		161.79^b^	70.96^b^	68.94^b^	14.19^b^	107.03^b^
0.1		156.04^b^	61.77^c^	77.48^a^	12.35^c^	90.92^c^
p-value		0.00	0.00	0.00	0.00	0.00
Interaction effects						
Soybean oil	0	226.37^a^	93.080^a^	57.09^c^	18.62^a^	187.89^a^
	0.05	167.66^c^	85.32^b^	68.26^b^	17.06^b^	116.46^c^
	0.1	164.60^c^	72.56^c^	70.39^b^	14.51^c^	108.72^cd^
Linseed oil	0	188.53^b^	66.95^d^	58.39^c^	13.39^d^	143.53^b^
	0.05	155.91^cd^	56.60^e^	69.62^b^	11.32^e^	97.60^d^
	0.1	147.49^d^	50.98^f^	84.57^a^	10.20^f^	73.11^e^
p-value		0.01	0.04	0.02	0.04	0.02
Pooled SEM		1.71	0.54	1.01	0.11	1.71

HDL, high-density lipoprotein; VLDL, very-low–density lipoprotein; LDL, low-density lipoprotein; PPE, pomegranate peel extract; SEM, standard error of the mean.

a–d Means within the same column carrying different superscripts are significantly different at p≤0.05.

**Table 8:** Effects of different dietary levels of pomegranate peel extract with different oil sources and their interactions on broiler breast muscle fatty acid profile (% of total fatty acids)

Items		Palmitic acid	Stearic acid	Oleic acid	Palmitoleic acid	Linoleic acid	Gamma-linolenic acid	Alpha-linolenic acid	Other fatty acids
Oils									
Soybean oil		22.09	5.60	34.83^a^	5.05^a^	26.16^a^	0.76^a^	1.46^b^	4.04^a^
Linseed oil		21.10	5.47	31.05^b^	4.71^b^	22.81^b^	0.62^b^	7.93^a^	3.31^b^
p-value		0.72	0.27	0.03	0.00	0.00	0.00	0.00	0.04
PPE level %									
0		21.49^b^	5.76	32.92	4.77^a^	24.83^b^	0.69^a^	4.50^a^	4.05^a^
0.05		20.91^a^	5.16	32.91	5.13^b^	25.20^a^	0.72^b^	4.81^b^	4.01^b^
0.1		22.40^c^	5.71	32.65	4.76^a^	23.41^c^	0.67^c^	4.77^c^	2.97^c^
p-value		0.00	0.32	0.22	0.08	0.00	0.01	0.001	0.033
Interaction effects									
Soybean oil	0	21.95	6.21	34.63^a^	4.56^ab^	26.06^ab^	0.78^a^	1.58^d^	4.22^a^
	0.05	20.93	5.00	34.61^a^	5.33^a^	27.10^a^	0.78^a^	1.62^d^	4.63^ab^
	0.1	22.00	5.60	34.25^a^	5.27^a^	25.30^b^	0.72^b^	1.18^e^	3.27^ab^
Linseed oil	0	21.02	5.30	31.20^b^	4.97^ab^	23.60^c^	0.60^c^	7.43^c^	3.88^ab^
	0.05	20.88	5.31	31.20^b^	4.93^ab^	23.30^c^	0.65^c^	8.00^b^	3.39^ab^
	0.1	21.40	5.81	31.04^b^	4.24^b^	21.52^d^	0.61^c^	8.36^a^	2.66^b^
p-value		0.004	0.31	0.03	0.002	0.03	0.042	0.00	0.035
Pooled SEM		0.11	0.18	0.15	0.07	0.18	0.01	0.03	0.18

PPE, pomegranate peel extract; SEM, standard error of the mean.

a–d Means within the same column carrying different superscripts are significantly different at p≤0.05.

**Table 9:** Effects of different dietary levels of pomegranate peel extract with different oil sources and their interactions on total phenol and total flavonoid contents of breast muscle (μg/g fresh weight) and lysozyme activity (μ/mL)

Items		Phenols	Flavonoids	Lysozymes
Oils				
Soybean oil		15.69^b^	2.13^b^	0.86^b^
Linseed oil		19.26^a^	4.28^a^	0.96^a^
p-value		0.00	0.00	0.01
PPE level %				
0		11.52^c^	1.70^c^	0.73^c^
0.05		14.27^b^	3.36^b^	0.90^b^
0.1		26.65^a^	4.55^a^	1.11^a^
p-value		0.00	0.00	0.00
Interaction effects				
Soybean oil	0	10.06^e^	0.42^f^	0.60^c^
	0.05	13.21^d^	2.36^e^	0.87^b^
	0.1	23.81^b^	3.60^c^	1.12^a^
Linseed oil	0	12.98^d^	2.99^d^	0.85^b^
	0.05	15.32^c^	4.35^b^	0.92^b^
	0.1	29.49^a^	5.50^a^	1.10^a^
p-value		0.007	0.02	0.005
Pooled SEM		0.22	0.07	0.02

PPE, pomegranate peel extract; SEM, standard error of the mean.

a–f Means within the same column carrying different superscripts are significantly different at p≤0.05.
